# Identification of Prognostic Signatures for Predicting the Overall Survival of Uveal Melanoma Patients

**DOI:** 10.7150/jca.30618

**Published:** 2019-08-27

**Authors:** Meijuan Xue, Jun Shang, Binglin Chen, Zuyi Yang, Qian Song, Xiaoyan Sun, Jianing Chen, Ji Yang

**Affiliations:** 1 Department of Dermatology, Zhongshan Hospital, Fudan University, Shanghai 200032, China.; 2 State Key Laboratory of Genetic Engineering, School of Life Sciences and Human Phenome Institute, Fudan University, Shanghai 200438, China.; 3 Department of Hematology, The First Affiliated Hospital of Soochow University, Shizijie Campus: NO.188, Shizijie Road, Suzhou 215006, P. R. China.; 4 Department of Medical Oncology, Fudan University Shanghai Cancer Center, 270 Dong-An Road, Shanghai 200032, China.; 5 Department of Hepatobiliary and Pancreatic Surgery, The First Affiliated Hospital of Zhengzhou University, Zhengzhou, Henan, China.

**Keywords:** Uveal melanoma, Prognosis, Gene mutations, Copy number variants

## Abstract

Uveal melanoma (UM) is an aggressive cancer which has a high percentage of metastasis and with a poor prognosis. Identifying the potential prognostic markers of uveal melanoma may provide information for early detection of metastasis and treatment. In this work, we analyzed 80 uveal melanoma samples from The Cancer Genome Atlas (TCGA). We developed an 18-gene signature which can significantly predict the prognosis of UM patients. Firstly, we performed a univariate Cox regression analysis to identify significantly prognostic genes in uveal melanoma (P<0.01). Then the glmnet Cox analysis was used to generate a powerful prognostic gene model. Further, we established a risk score formula for every patient based on the 18-gene prognostic model with multivariate Cox regression. We stratified patients into high- and low-risk subtypes with median risk score and found that patients in high-risk group had worse prognosis than patients in low-risk group. Multivariate Cox regression analysis demonstrated that 18-gene model risk score was independent of clinical prognostic factors. We identified four genes whose mutations were closely to UM patients' prognosis or risk score. We also explored the relationship between copy number variation and risk score and found that high risk group showed more chromosome aberrations than low risk group. Gene Set Enrichment Analysis (GSEA) analysis showed that the different biological pathways and functions between low and high risk group. In summary, our findings constructed an 18-gene signature for estimating overall survival (OS) of UM. Patients were categorized into two subtypes based on the risk score and we found that high risk group showed more chromosome aberrations than low risk group.

## Introduction

Uveal melanoma, the most common primary intraocular malignancy, mainly has an increased risk of liver metastatic involvement. Though effective therapy, surgical enucleation and radiotherapy have improved local control, however up to 50% patients will eventually die of their disease 1, 2. Uveal melanoma has been considered with the worse prognosis and poorer response to chemotherapy compared with cutaneous melanoma. Therefore, identifying the potential prognostic markers of uveal melanoma may provide information for early detection of recurrence and treatment. Although many studies have identified some important genes and pathways in the diagnosis and treatment of uveal melanoma, the prognosis of uveal melanoma patients was still very poor 3, 4. Hence, it's urgent to unveil new markers to evaluate their prognosis.

Nowadays, there are lots of emerging high-throughput sequencing technologies and databases for development of diagnostic and prognostic signatures of cancer. Although a 15-gene expression panel of UM patients was explored to determine the risk of metastasis, the signature for prognostic results remained to be further elucidated 5. Gene mutations and chromosome copy number variants strongly correlated with UM outcome. UM patients also display a pattern of mutations. GNAQ and GNA11 mutations are reported to promote cell proliferation and metastasis 6. BAP1 mutations are related to metastasis and EIF1AX mutations are associated with favorable metastatic-free survival 7-10. Uveal melanoma patients with chromosome 3 copy number loss are associated with a high risk of metastasis and a poor outcome 11, 12. It was reported that UM tumor progression typically displays chromosomal abnormalities. UM patients with chromosome 3 loss, 8q gain and 1p loss have reduced overall survival 12. Therefore, exploring the gene mutations and copy number variations may provide insights into uveal melanoma prognosis.

In the present paper, we conducted uveal melanoma data by using the publicized data from the TCGA database. By using glmnet Cox model and Cox regression analysis, we developed an 18-gene prognostic model to demonstrate the association between 18-gene model and prognostic power of uveal melanoma. Meantime, we also found the specific gene mutations and chromosome copy number variations involved in uveal melanoma associated with overall survival. By utilizing 18-gene prognostic model, gene mutations and copy number variations of UM may provide evidence for the selection and determination of an individualized and targeted therapeutic treatment for each patient.

## Materials and Methods

### Uveal melanoma data collection and analysis from TCGA

We retrospectively collected uveal melanoma gene expression data and corresponding clinical information of TCGA from UCSC Xena Public Data Hubs (https://xena.ucsc.edu/public-hubs/). All expression values were transformed with log2 (FPKM+1). Copy number profiles from Affymetrix Genome-Wide Human SNP Array 6.0 platform and segments were mapped to hg38 genome assembly (https://xena.ucsc.edu/public-hubs/). Mutation data containing somatic variants was stored in the form of Mutation Annotation Format (MAF) and was downloaded from Genomic Data Commons (GDC) (https://portal.gdc.cancer.gov/). There were 80 patients in our analysis.

### Construction of a prognostic gene model

Univariable Cox regression analysis was performed to identify gene which was considered statistically significant if its p value was less than or equal to 0.01. We further used glmnet Cox model to trained these identified genes for 2000 times independently (R package, glmnet, version 2.0-16), and finally selected a best suitable prognostic model with the highest frequency 13, 14. With this prognostic gene model, the prognostic genes were strictly included. Subsequently, a risk score was established using the sum of each prognostic gene expression values weighted by their regression coefficients as previously described 15, 16. Based on this formula, the risk score for each patient was calculated. Then the uveal melanoma cohort was separated into high- and low- risk groups using the median risk score as a cut-off 17, 18. N represented the number of prognostic model genes, Coei was the regression coefficient value of each gene and Expi represented the expression of prognostic model genes.

Risk Score = 
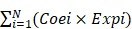


### Gene mutations and copy number variants analysis

The significant mutation genes reported in uveal melanoma were used to investigate the differences between high and low risk group 9. The relationship between risk score and chromosomal copy number variations (CNVs) were explored. The R package 'copynumber' was used to show the CNVs according the order of risk scores 19.

### Functional enrichment analysis of prognostic gene model

The Gene Ontology (GO) terms and Kyoto Encyclopedia of Genes and Genomes (KEGG) pathways between high and low risk group were identified by The Gene Set Enrichment Analysis (GSEA) (https://pypi.org/project/gseapy/) 20.

### Statistical analysis

The time-independent receiver operating characteristic curve (ROC) was to assess the risk prediction power of the prognostic gene model for overall survival (R package, survivalROC, version 1.0.3). Kaplan-Meier survival analysis together with a log-rank test was implemented to compare the difference of overall survival between high- and low-risk cohort using R package “survival” 21. R package “glmnet” was used to perform Cox proportional hazards regression with the least absolute shrinkage (glmnet, version 2.0-16). Multivariate Cox regression analysis was performed to provide evidence that the prognostic gene model was independent of other clinicopathological factors 22. All analysis was performed with R software (version 3.3.0). P<0.05 was considered statistically significant.

## Results

### Construction of an 18-gene prognostic model

**Figure 1:**
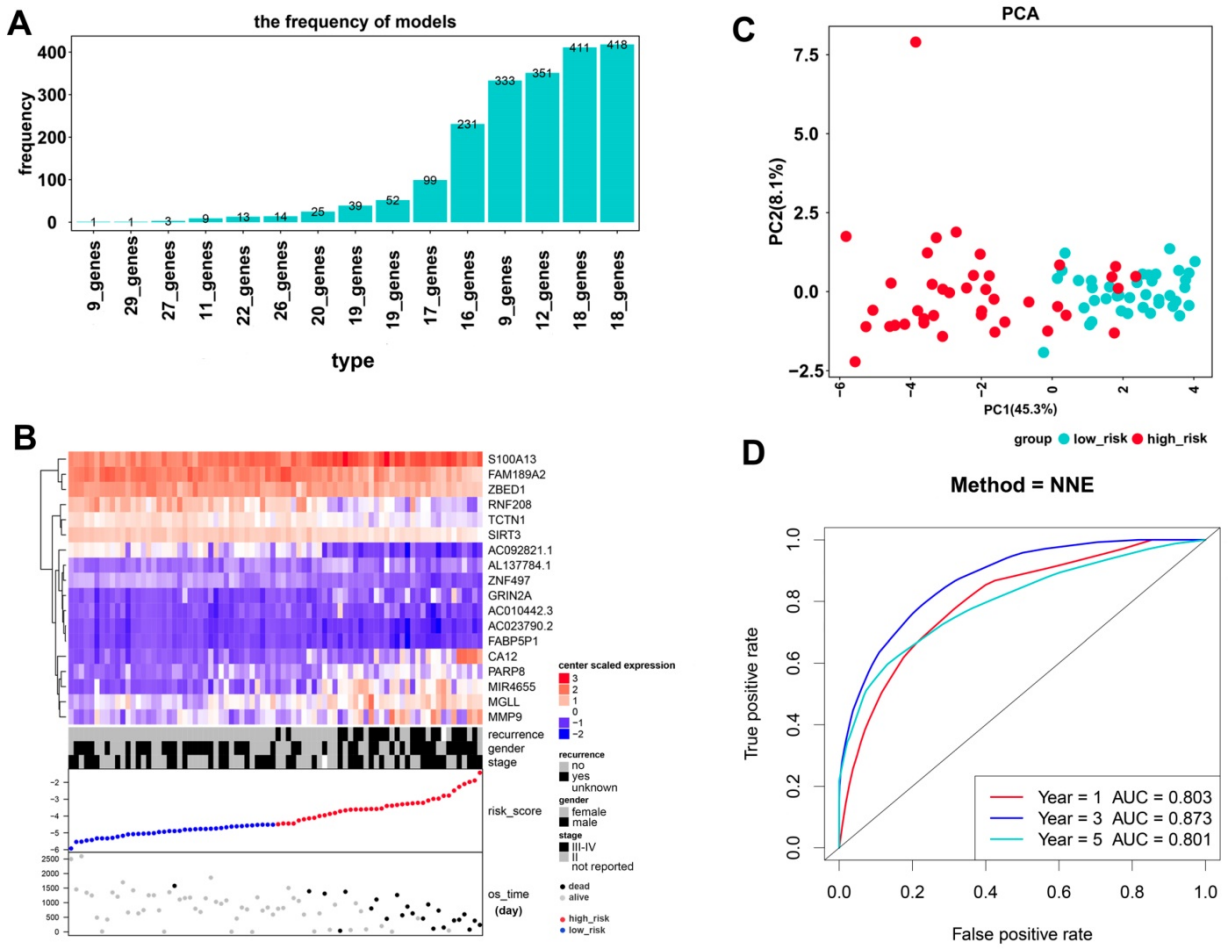
Construction of an 18-gene prognostic model. **(A)** The results of prognostic gene models trained by glmnet cox regression method and ranked by the frequency of occurrence. **(B)** The expression distribution of the 18-gene signature according to patients' risk score. The risk scores for all patients in uveal melanoma were plotted in ascending order and marked as low risk (blue) or high risk (red). The survival status of the patients were marked as dead (black) and alive (grey). Almost all recurrence patients were in high risk group.** (C)** Principal components analysis (PCA) of the dataset for high-risk and low-risk samples for uveal melanoma. The high-risk samples were marked by red dots and the low-risk samples were marked by blue dots. **(D)** Receiver operating characteristic (ROC) plot illustrated the prediction efficacy of the 18-gene prognostic model. An area of under curve for one year, three years and five years were 0.803, 0.873 and 0.801, separately.

**Figure 2:**
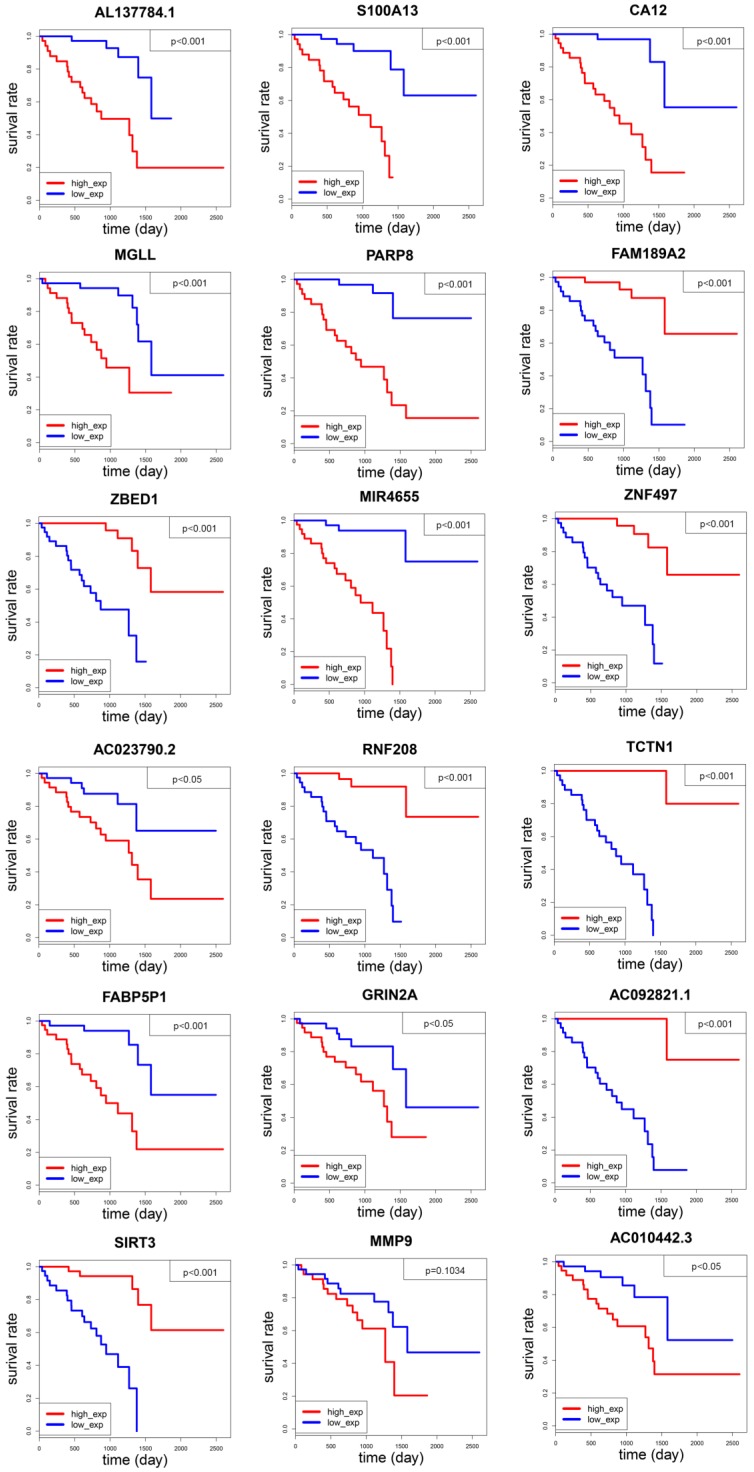
Kaplan-Meier overall survival (OS) curves of the 18 genes within our prognostic genes. The expression of FAM189A2, ZBED1, ZNF497, RNF208, TCTN1, AC092821.1 and SIRT3 was positively associated with OS of UM patients; the expression of genes including AL137784.1, S100A13, CA12, MGLL, PARP8, MIR4655, AC023790.2, FABP5P1, GRIN2A and AC010442.3 was negatively related to the overall survival of UM patients; the expression of MMP9 played no significance in overall survival of UM patients.

As a result, 4388 significantly prognostic genes in 80 uveal melanoma samples were identified by using univariate Cox regression analysis (p ≤0.01). And then a Cox proportional hazards regression model from glmnet was used to select the key genes played important roles in the prognosis of patients. Ten-fold cross-validation for the Cox model was performed to estimate the penalty parameter to minimize the risk of overfitting. After 2000 iterations, we ranked fifteen significant prognostic gene models according to their frequency (Figure 1A, Supplementary 1). We selected the one with the highest frequency as our final prognosis gene model, including 18 genes, of which are S100A13, FAM189A2, ZBED1, RNF208, TCTN1, SIRT3, AC092821.1, AL137784.1, ZNF497, GRIN2A, AC010442.3, AC023790.2, FABP5P1, CA12, PARP8, MIR4655, MGLL, and MMP9 (Figure 1B, Table 1). Kaplan-Meier survival curves analysis of the 18 genes for overall survival of uveal melanoma were shown in Figure 2. We further calculated a risk score for each patient in uveal melanoma samples with the risk score formula. Then patients were divided into high- and low-risk groups using the median risk score value as cut-off to investigate the prognostic role of the 18-gene signature model in overall survival. Next, principal components analysis (PCA) based on the 18 gene expression demonstrated a different separation pattern between high- and low-risk group (Figure 1C), indicating distinct phenotypes among uveal melanoma. Further, ROC curve was performed to assess the prognostic efficiency of the model. As a prediction figure, the final model of prediction power for one year, three years and five years achieved an area of under curve of 0.803, 0.873 and 0.801, separately (Figure 1D). Utilizing our predictive model of uveal melanoma, we could achieve early prediction of distinguishing poor and good prognosis of patients.

### Prognostic role of an 18-gene model in uveal melanoma

**Figure 3:**
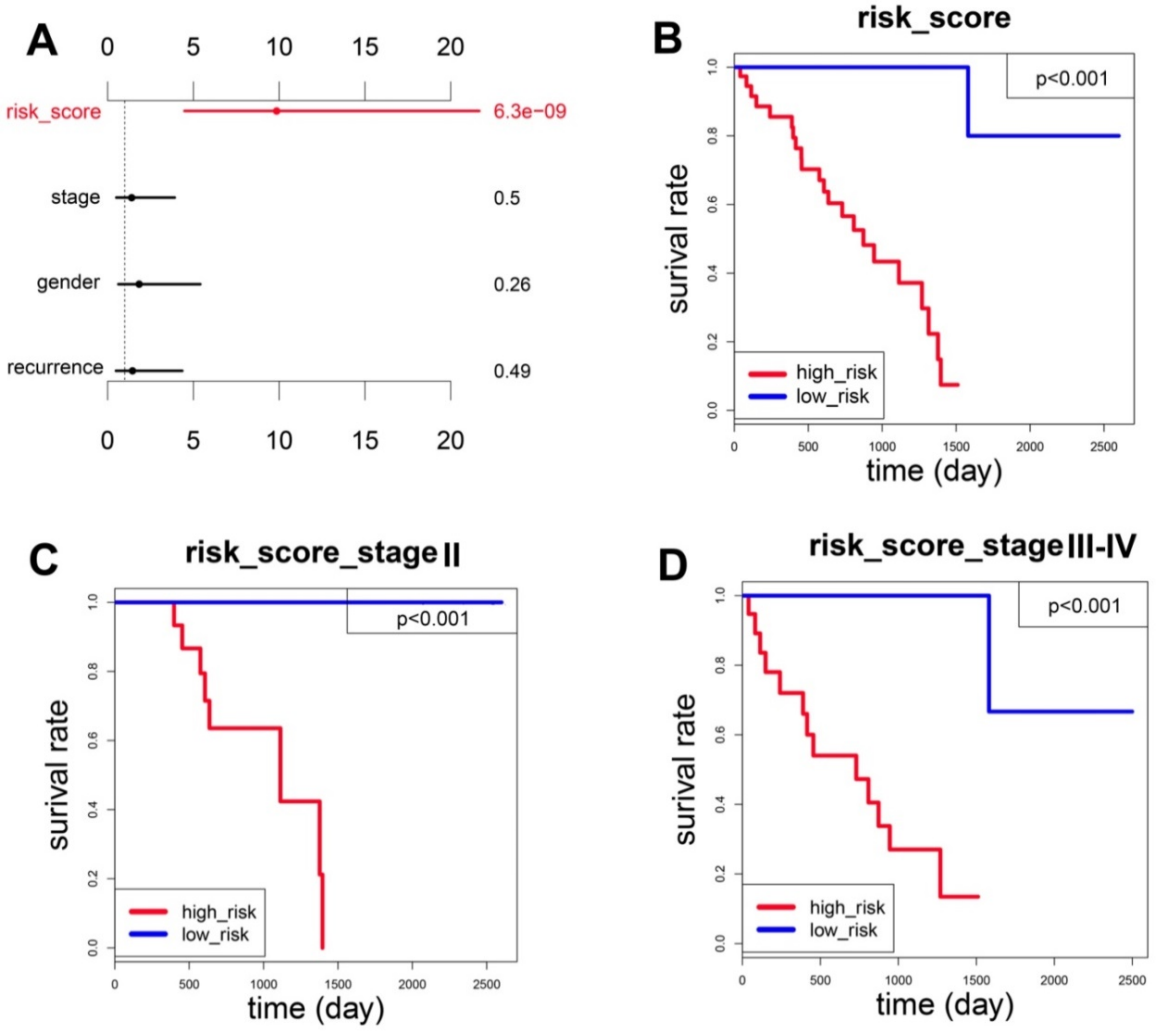
The 18-gene prognostic model predicted overall survival of patients with uveal melanoma.** (A)** Multivariate Cox regression analysis showing the risk score of the 18-gene signature serve as an independent prognostic factor in uveal melanoma. **(B)** Kaplan-Meier survival curves of overall survival (OS) between high-and low-risk patients in the UM cohort. **(C-D)** The Kaplan-Meier curve of overall survival in the stage II, stage III-IV cohort stratified by 18-gene signature in high and low risk.

After constructing an 18-gene prognostic model, we further performed a multivariate Cox regression analysis to confirm its power for independently predicting prognosis. The results showed that the 18-gene model risk score was independent of stage, gender, and recurrence for overall survival in UM patients (HR=9.298, 95%CI=4.381-19.734, P<0.001, Figure 3A, Table 2). The hazard ratio (HR) value of risk score is greater than one, also much greater than the HR values of stage, gender, and recurrence, which means that UM patients whose risk score is high will have a worse prognosis. After separating UM patients into high- and low-risk groups, we sought to investigate the correlation between two groups and overall survival of uveal melanoma. The Kaplan-Meier curve demonstrated that uveal melanoma patients with high risk score had poorer survival than those with low risk score patients (log-rank test, p<0.001) (Figure 3B). Consistently with the patients observed in the high-risk group had higher recurrence rate and larger dead numbers compared with low-risk group (Figure 1B). What's more, we also investigated the 18-gene prognostic signature in UM patients at pathological stage II and stage III-IV. As a result, the 18-gene signature model could predict UM patients with different prognosis in different subgroups including pathological stage II, stage III-IV (Figure 3C, 3D).

### Identification of gene mutations and copy number variants in uveal melanoma

**Figure 4:**
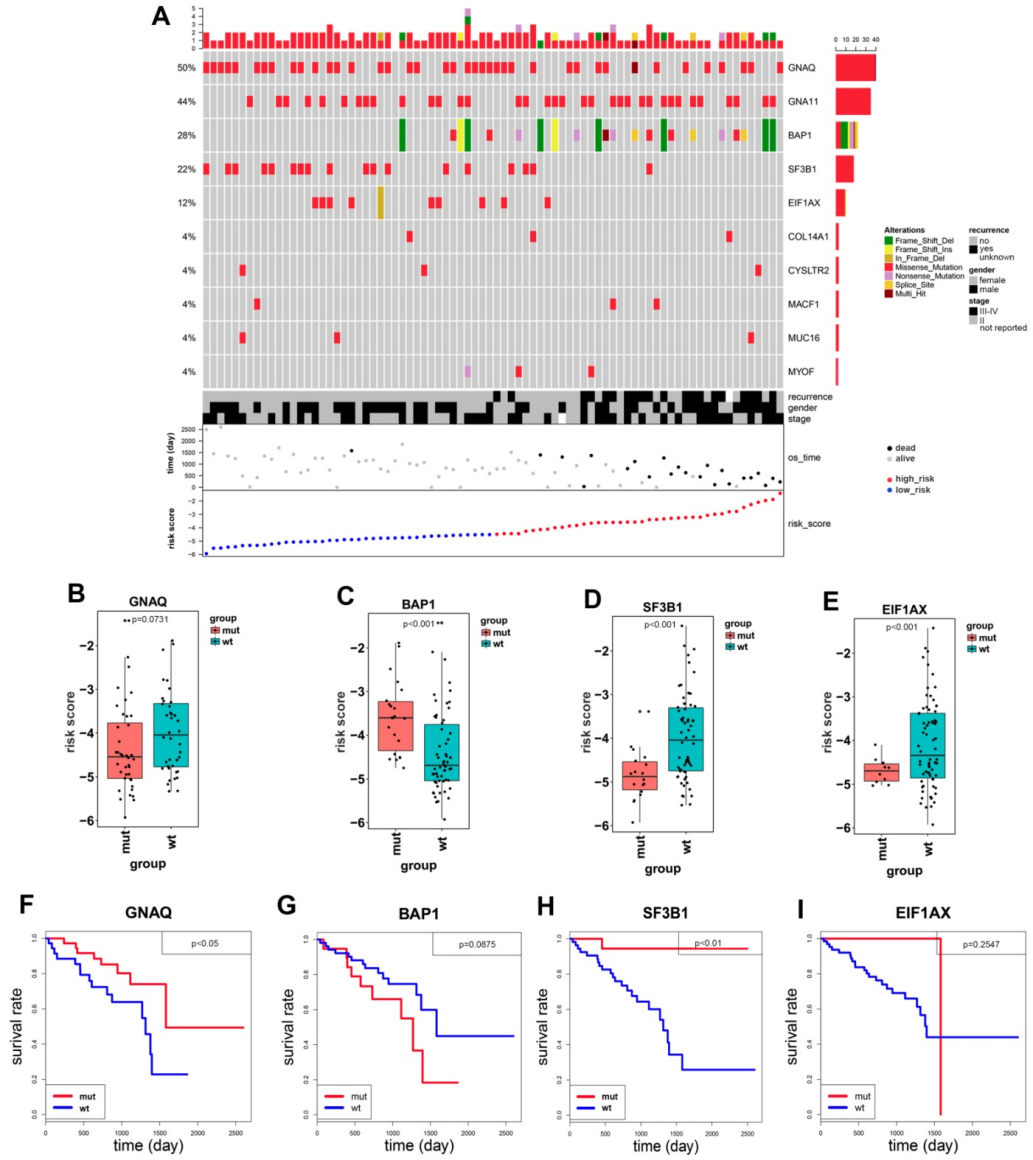
Identification and prognostic role of gene mutations in uveal melanoma patients. **(A)** The mutation profile distribution of 10 significant mutation genes based on patients' risk score. The different colors represent the different patterns of the mutation. **(B-E)** The mutated and wildtype genes of patients in the risk score boxplot graph (GNAQ, BAP1, SF3B1 and EIF1AX). The graph demonstrated the median risk score and confidence interval. **(F-I)** The Kaplan-Meier curves of overall survival in the GNAQ, BAP1, SF3B1 and EIF1AX cohort stratified by gene mutations and wildtypes, separately.

Uveal melanoma displays gene mutations and copy number variations that correlate strongly with clinical outcome which are not present in cutaneous melanoma. Therefore, we investigated if there are gene mutation differences between high- and low-risk groups. Ten significantly mutated genes were detected: GNAQ, GNA11, BAP1, SF3B1, EIF1AX, COL14A1, CYSLTR2, MACF1, MUC16 and MYOF. As shown in Figure 4A, patients with SF3B1 mutations and EIF1AX mutation were mainly distributed in low-risk group, while patients with BAP1 mutations mainly emerged in high-risk group. BAP1 mutations were identified in 28% uveal melanoma patients, and the types of mutations included frame shift deletion, missense mutation, nonsense mutation, splice site and frame shift insert. SF3B1 and EIF1AX mutations are mainly missense mutations.

**Figure 5:**
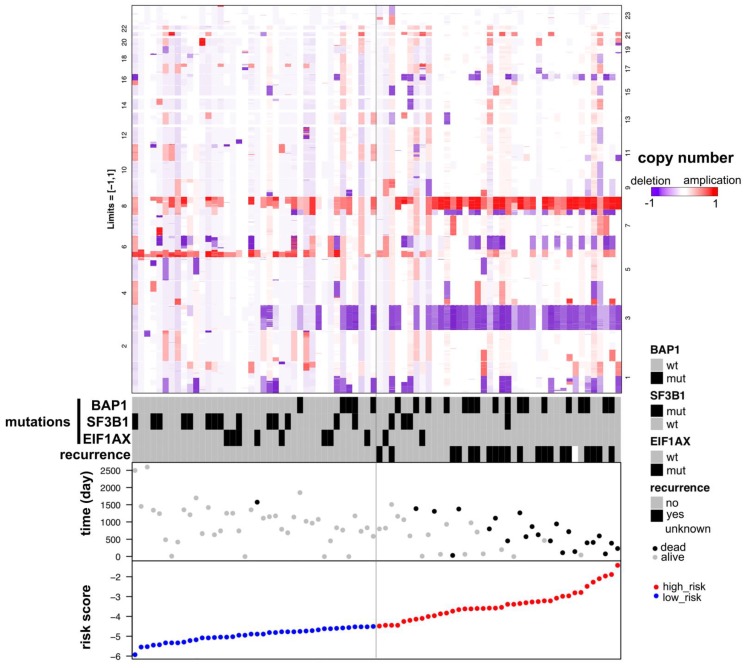
Distribution of copy number variations (CNVs) according to the risk score of patients with uveal melanoma. The numbers on two columns represent the ranked number of the chromosome. UM patients in high-risk group have much more chromosome 3 deletions and chromosome 8 applications.

Patients with SF3B1 and EIF1AX wildtypes have higher risk score than patients with mutation types (Figure 4D, 4E). The Kaplan-Meier curve showed that the overall survival in SF3B1 mutation group was significantly longer than in wildtype group (p=0.0071, Figure 4H), while the overall survival of EIF1AX in mutation cohort versus wildtype cohorts had no statistical difference (p=0.2547, Figure 4I). BAP1 risk score was much higher in mutation group than in wildtype group (p=6×10^-4^, Figure 4C), adversely compared to GNAQ, SF3B1, and EIF1AX. Notably, there was no significantly prognostic difference between BAP1 mutated group and wildtype group (p=0.0875, Figure 4G). Meanwhile, we found that patients with GNAQ mutation have more favorable prognosis than patients in GNAQ wildtype group (p=0.0426, Figure 4B, 4F). As shown in Figure 5, we found that UM patients in high-risk group have much more chromosome 3 deletion and chromosome 8 gain. These specific gene mutations and chromosome copy number variations (CNVs) can also be a prognostic biomarker for uveal melanoma patients.

### Biological process identified by Functional enrichment analysis

**Figure 6:**
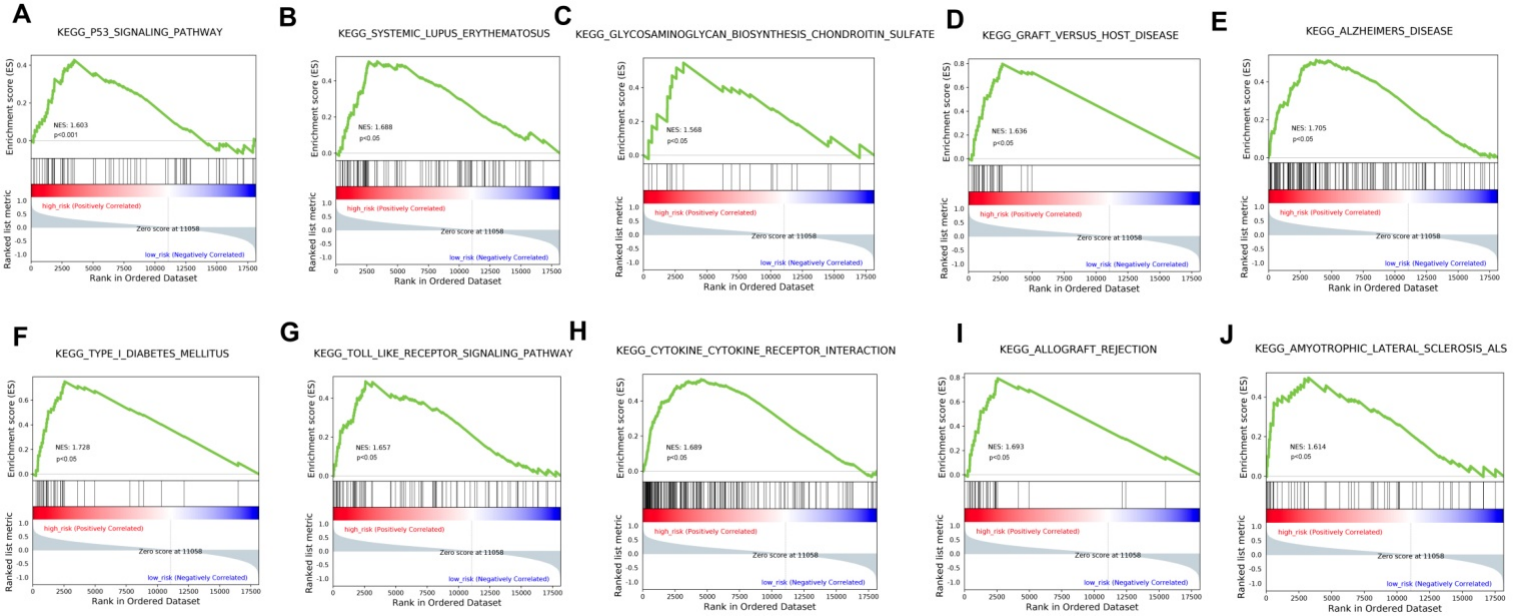
GSEA analysis of the KEGG pathway enrichment in high-risk versus low-risk patients with uveal melanoma.

**Figure 7:**
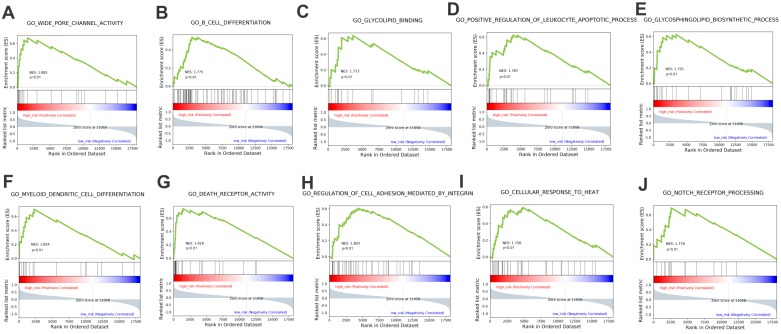
GSEA analysis of the GO terms enrichment in high-risk patients compared with the low-risk ones.

The stratification power of the 18-gene prognostic model in predicting overall survival of UM could be ascribed to their roles in tumor development and metastasis progress. Therefore, GSEA analysis was performed to identify associated enriched genes or pathways in datasets. We selected top 10 ranked KEGG sets and GO sets results shown in Figure 6 and Figure 7. In the results, we found that the “p53 signaling pathway” were enriched in the high-risk group patients. Several studies have investigated this pathway is associated with the prognosis of uveal melanoma. Young Sun et al. demonstrated that p53 signaling pathway played a major role in mediating cellular response to UM radiation-induced DNA damage and may be defective in UM. Further, other cancer related pathways and genes such as “Toll-like receptor signaling pathway”, “cytokine receptor interaction”, “wide pore channel activity”, “B cell differentiation”, “death receptor activity”, “regulation of cell adhesion mediated by integrin”, “cellular response to heat” and “Notch receptor processing”, were enriched in high-risk UM groups. In conclusion, functional enrichment analysis results showed that this 18-gene prognostic model may play a significant role in UM development and biological progress.

## Discussion

In our study, we used univariate Cox regression analysis and glmnet Cox analysis method to deal with the 80 uveal melanoma samples from the TCGA. As a result, we identified an 18-gene prognostic model, including S100A13, FAM189A2, ZBED1, RNF208, TCTN1, SIRT3, AC092821.1, AL137784.1, ZNF497, GRIN2A, AC010442.3, AC023790.2, FABP5P1, CA12, PARP8, MIR4655, MGLL, and MMP9 (Figure 1B, Table 1). As we know, there were several known cancer-specific biomarkers of the model that had already been translated into clinical prognostic signatures. For example, low levels or no expression of S100A13 may be one of the key predictive markers to identify melanoma patients responding to dacarbazine\temozolomide chemotherapy 23. Daniela et al. demonstrated S100A13 as a new angiogenic indicator that facilitates human UM progression and as a promising prognostic marker 24. Jasmine et al. showed that SIRT3 overexpression played a pro-proliferative function in melanoma and was essential for melanoma growth and survival 25. Chen et al. revealed that MMP9 played vital roles in facilitating the metastasis of uveal melanoma cells and the involvement of MMPS in cancer progression has been reported in various cancer cell types 26-28. Moreover, Harbour et al. presented a 15-gene signature that could distinguish UM patients between two groups with high risk and low risk of metastasis without regarding to copy number variations and gene mutation status 5. A. Gordon Robertson et al. performed a comprehensive multi-platform analysis of 80 cases of primary uveal melanoma from the Cancer Genome Atlas, finally they identified and characterized four different subtypes with unique genomic aberrations, transcriptional features and clinical outcomes 9. Their study mainly aimed at revealing distinct molecular and clinical UM profiles, emphasizing the need for stratified UM patient management. While our study built up a prognostic gene model to predict the overall survival of UM patients, aiming at patients' prognosis but not molecular subtypes. Although we also investigated the gene mutations and copy number variations of UM patients. However, importantly, we analyzed them in order to find if there were different gene mutations and copy number variations between high- and low-risk cohorts based on the 18-gene prognostic model, rather than exploring their relationships with UM prognosis independently. If more gene mutations and copy number variations exist in high-risk group consistent with the previous study, which show our prognostic gene model is more accurate. In our work, we first demonstrated an 18-gene signature could act as prognostic signatures for uveal melanoma, which could provide insights into new prognostic biomarkers exploration. Multivariate Cox regression analysis showed that the 18-gene risk score can be an independent prognostic factor.

Uveal melanoma displays gene mutations and copy number variations that correlate strongly with clinical outcome. Therefore, we investigated if there were gene mutation differences between high- and low-risk groups of UM patients. In the 80 uveal melanoma samples, 50% had GNAQ and 44% had GNA11. In our study, most of uveal melanoma harbored mutations in GNAQ, GNA11 as previously reported 9, 29, 30. These gene mutations were not identified in the cutaneous melanoma, which was consistent with the literature 31. Mutations in GNAQ (50%) and GNA11 (44%) are early events that promote cell proliferation, suggesting that activated G-protein signaling plays a crucial role in early uveal melanoma development 12. In addition to mutated genes mentioned above, uveal melanoma also harbored additional SF3B1 (22%), EIF1AX (12%), COL14A1 (4%), CYSLTR2 (4%), MACF1 (4%), MUC16 (4%) and MYOF (4%). BAP1, SF3B1 and EIF1AX gene mutations in UM patients are related to poor, median and favorable prognosis, respectively 7, 8, 32. While our study identified UM patients to have a favorable prognosis harboring GNAQ and SF3B1 mutations (Figure 4F, 4H). BAP1 inactivation being related to poor prognosis in uveal melanoma is well studied 9, 33, 34. However, In our Kaplan-Meier analysis, the difference in overall survival among patients with BAP1 mutation and patients without mutation was not statistically significant (P = 0.0875), indicating that BAP1 mutation was not significantly associated with OS (Figure 4G). This discrepancy may be contributed to the small number of UM patients of the study. Except for exploring related gene mutations with UM overall survival, we also investigated chromosome copy number variants between high- and low-risk groups. Our result showed that UM patients with chromosome 3 loss and chromosome 8 gain should be considered in high-risk group. Consistently, Scholes et al. demonstrated that half uveal melanoma patients with loss copy of chromosome 3, combining with other chromosome variations and clinical information, such as 6p and 8q gain, which will largely improve prognostic accuracy 11, 35, 36. Prior studies have also shown poorer clinical outcomes to be associated with higher chromosome 8q copy number 37-39.

Biological pathway profiling showed that tumorigenesis and progress related processes such as p53 signaling, Notch receptor signaling, regulation of cell adhesion mediated by integrin and other pathways were enriched in high-risk UM patients. The results implied that specific strategies targeted these biological pathways may achieve therapeutic efficacy in high-risk UM group of poor prognosis. Further clinical studies on these pathways are needed.

In summary, we identified an 18-gene prognostic model associated with UM overall survival, combining with determination of chromosome copy number variants and gene mutations can be a useful tool to predict UM patient's prognosis. However, several limitations to this study exist. First, our study is retrospective, and prospective study should be further validated. Second, we just constructed our model in one cohort owing to the small number of UM samples. Validating this 18-gene prognostic model in a larger cohort of uveal melanoma patients can make the prognosis more convincing.

## Supplementary Material

Supplementary table.

## Figures and Tables

**Table 1:** Univariate and multivariate Cox analysis for overall survival of 18 risk genes in our prognostic gene model.

genes	Univariate cox regression			Multivariate cox regression		
	HR	95%CI	p value	HR	95%CI	p value
AL137784.1	13.931	4.166-46.587	<0.001	10.377	2.442-44.091	0.002
stage				2.434	0.851-6.961	0.097
gender				3.59	1.274-10.113	0.016
recurrence				6.585	2.148-20.187	0.001
S100A13	6.607	2.829-15.43	<0.001	3.674	1.276-10.575	0.016
stage				1.582	0.617-4.057	0.34
gender				1.854	0.697-4.932	0.216
recurrence				4.811	1.475-15.687	0.009
CA12	2.07	1.621-2.644	<0.001	1.752	1.299-2.362	<0.001
stage				1.732	0.678-4.425	0.251
gender				1.18	0.39-3.573	0.77
recurrence				5.368	1.793-16.073	0.003
MGLL	1.811	1.387-2.365	<0.001	1.344	0.951-1.899	0.093
stage				0.922	0.346-2.455	0.87
gender				1.861	0.664-5.215	0.238
recurrence				7.306	2.469-21.62	<0.001
PARP8	4.458	2.308-8.609	<0.001	2.437	1.078-5.512	0.032
stage				0.94	0.37-2.388	0.897
gender				2.461	0.921-6.579	0.073
recurrence				5.323	1.57-18.041	0.007
FAM189A2	0.1	0.041-0.246	<0.001	0.193	0.061-0.61	0.005
stage				1.07	0.424-2.696	0.886
gender				1.958	0.725-5.288	0.185
recurrence				2.869	0.719-11.457	0.136
ZBED1	0.144	0.063-0.329	<0.001	0.208	0.072-0.598	0.004
stage				1.375	0.537-3.521	0.507
gender				2.643	0.943-7.407	0.065
recurrence				5.037	1.684-15.063	0.004
MIR4655	2.413	1.66-3.507	<0.001	2.219	1.18-4.17	0.013
stage				0.623	0.213-1.824	0.388
gender				2.725	1.004-7.398	0.049
recurrence				3.442	0.93-12.737	0.064
ZNF497	0.002	0-0.032	<0.001	0.001	0-0.029	<0.001
stage				0.885	0.353-2.217	0.794
gender				3.513	1.136-10.862	0.029
recurrence				4.213	1.586-11.193	0.004
AC023790.2	5.46E+08	inf	<0.001	9129374	1192.056-inf	<0.001
stage				0.993	0.395-2.492	0.988
gender				1.75	0.638-4.803	0.277
recurrence				7.389	2.649-20.609	<0.001
RNF208	0.2	0.097-0.414	<0.001	0.239	0.107-0.534	<0.001
stage				1.383	0.555-3.447	0.486
gender				2.692	0.956-7.581	0.061
recurrence				2.959	1.029-8.507	0.044
TCTN1	0.072	0.023-0.223	<0.001	0.147	0.037-0.594	0.007
stage				1.19	0.477-2.967	0.709
gender				1.964	0.743-5.194	0.174
recurrence				2.83	0.787-10.174	0.111
FABP5P1	9463.164	280.209-inf	<0.001	643.249	10.698-inf	0.002
stage				1.148	0.466-2.828	0.765
gender				2.118	0.792-5.665	0.135
recurrence				6.091	2.079-17.843	0.001
GRIN2A	3.63	2.082-6.328	<0.001	2.283	1.187-4.39	0.013
stage				1.66	0.633-4.352	0.303
gender				2.171	0.809-5.828	0.124
recurrence				7.341	2.507-21.495	<0.001
AC092821.1	0.138	0.051-0.371	<0.001	0.233	0.083-0.657	0.006
stage				1.073	0.428-2.687	0.881
gender				1.773	0.659-4.766	0.257
recurrence				3.202	1.041-9.853	0.042
SIRT3	0.013	0.003-0.061	<0.001	0.025	0.003-0.177	<0.001
stage				1.893	0.722-4.962	0.194
gender				2.295	0.852-6.181	0.1
recurrence				3.14	1.001-9.854	0.05
MMP9	2.105	1.587-2.791	<0.001	1.707	1.227-2.374	0.001
stage				0.594	0.211-1.675	0.324
gender				1.857	0.687-5.016	0.222
recurrence				6.242	2.128-18.307	0.001
AC010442.3	16.78	3.688-76.341	<0.001	10.194	1.715-60.596	0.011
stage				0.906	0.355-2.311	0.836
gender				3.832	1.304-11.259	0.015
recurrence				7.163	2.445-20.982	<0.001

**Table 2:** Univariate and multivariate Cox regression analysis for overall survival of 18 gene risk score and other clinicopathological factors.

Terms	Univariate cox regression			Multivariate cox regression		
	HR	95%CI	P value	HR	95%CI	p value
risk score	10.194	4.95-20.993	<0.001	9.298	4.381-19.734	<0.001
Stage	1.396	0.578-3.372	0.459	1.408	0.517-3.835	0.503
Gender	1.688	0.678-4.203	0.261	1.839	0.639-5.291	0.259
Recurrence	8.851	3.368-23.26	<0.001	1.454	0.497-4.258	0.494

## Abbreviations

UM: uveal melanoma
TCGA: The Cancer Genome Atlas
GSEA: Gene Set Enrichment Analysis
OS: overall survival
CNVs: copy number variations
FPKM: Fragments Per Kilobase Million
MAF: Mutation Annotation Format
GDC: Genomic Data Commons
GO: Gene Ontology
KEGG: Kyoto Encyclopedia of Genes and Genomes
ROC: receiver operating characteristic curve
