# Supplementary material for: Identification of Prognostic Signatures for Predicting the Overall Survival of Uveal Melanoma Patients
Source: J Cancer. 2019 Aug 27;10(20):4921–31. doi: 10.7150/jca.30618 (PMC6775505; doi:10.7150/jca.30618)
Supplement: Supplementary file 1 — Supplementary table. [file jcav10p4921s1.pdf]

Tables S1: A list of fifteen detailed gene prognostic models.

| type | genes | frequency |
|---|---|---|
| 9_genes | ENSG00000119139.15;ENSG00000074410.12;ENSG00000135063.16;ENSG00000174586.9;ENSG00000143036.15;ENSG00000204852.14;ENSG00000214776.8;ENSG00000142082.13;ENSG00000100196.9 | 1 |
| 29_genes | ENSG00000211941.3;ENSG00000183624.12;ENSG00000213856.3;ENSG00000219755.1;ENSG00000176177.9;ENSG00000234253.1;ENSG00000281205.1;ENSG00000183578.5;ENSG00000189171.12;ENSG00000074410.12;ENSG00000074416.12;ENSG00000244245.1;ENSG00000167393.15;ENSG00000170469.9;ENSG00000103154.8;ENSG00000214717.8;ENSG00000174586.9;ENSG00000255621.1;ENSG00000240622.1;ENSG00000250492.1;ENSG00000228887.3;ENSG00000229481.2;ENSG00000154479.11;ENSG00000236972.2;ENSG00000183454.12;ENSG00000142082.13;ENSG00000106178.5;ENSG00000166415.13;ENSG00000250645.1 | 1 |
| 27_genes | ENSG00000211941.3;ENSG00000183624.12;ENSG00000213856.3;ENSG00000219755.1;ENSG00000176177.9;ENSG00000281205.1;ENSG00000189171.12;ENSG00000074410.12;ENSG00000074416.12;ENSG00000244245.1;ENSG00000167393.15;ENSG00000170469.9;ENSG00000103154.8;ENSG00000214717.8;ENSG00000174586.9;ENSG00000255621.1;ENSG00000219797.2;ENSG00000240622.1;ENSG00000250492.1;ENSG00000228887.3;ENSG00000229481.2;ENSG00000154479.11;ENSG00000236972.2;ENSG00000183454.12;ENSG00000142082.13;ENSG00000166415.13;ENSG00000250645.1 | 3 |
| 11_genes | ENSG00000119139.15;ENSG00000074410.12;ENSG00000151883.15;ENSG00000135063.16;ENSG00000214717.8;ENSG00000265089.1;ENSG00000174586.9;ENSG00000143036.15;ENSG00000204852.14;ENSG00000214776.8;ENSG00000142082.13 | 9 |
| 22_genes | ENSG00000183624.12;ENSG00000213856.3;ENSG00000219755.1;ENSG00000176177.9;ENSG00000281205.1;ENSG00000189171.12;ENSG00000074410.12;ENSG00000074416.12;ENSG00000244245.1;ENSG00000170469.9;ENSG00000214717.8;ENSG00000174586.9;ENSG00000255621.1;ENSG00000219797.2;ENSG00000228887.3;ENSG00000229481.2;ENSG00000154479.11;ENSG00000236972.2;ENSG00000183454.12;ENSG00000214776.8;ENSG00000142082.13;ENSG00000250645.1 | 13 |

| | | |
|---|---|---|
| 26_genes | ENSG00000183624.12;ENSG00000213856.3;ENSG00000219755.1;ENSG00000176177.9;ENSG00000281205.1;ENSG00000189171.12;ENSG00000074410.12;ENSG00000074416.12;ENSG00000244245.1;ENSG00000167393.15;ENSG00000170469.9;ENSG00000103154.8;ENSG00000214717.8;ENSG00000174586.9;ENSG00000255621.1;ENSG00000219797.2;ENSG00000240622.1;ENSG00000228887.3;ENSG00000229481.2;ENSG00000154479.11;ENSG00000236972.2;ENSG00000183454.12;ENSG00000214776.8;ENSG00000142082.13;ENSG00000166415.13;ENSG00000250645.1 | 14 |
| 20_genes | ENSG00000183624.12;ENSG00000219755.1;ENSG00000176177.9;ENSG00000281205.1;ENSG00000189171.12;ENSG00000074410.12;ENSG00000074416.12;ENSG00000244245.1;ENSG00000170469.9;ENSG00000214717.8;ENSG00000174586.9;ENSG00000255621.1;ENSG00000219797.2;ENSG00000229481.2;ENSG00000154479.11;ENSG00000236972.2;ENSG00000183454.12;ENSG00000214776.8;ENSG00000142082.13;ENSG00000250645.1 | 25 |
| 19_genes | ENSG00000183624.12;ENSG00000219755.1;ENSG00000176177.9;ENSG00000281205.1;ENSG00000189171.12;ENSG00000074410.12;ENSG00000074416.12;ENSG00000244245.1;ENSG00000170469.9;ENSG00000214717.8;ENSG00000174586.9;ENSG00000255621.1;ENSG00000229481.2;ENSG00000154479.11;ENSG00000236972.2;ENSG00000183454.12;ENSG00000214776.8;ENSG00000142082.13;ENSG00000250645.1 | 39 |
| 19_genes | ENSG00000183624.12;ENSG00000219755.1;ENSG00000176177.9;ENSG00000281205.1;ENSG00000189171.12;ENSG00000074410.12;ENSG00000074416.12;ENSG00000244245.1;ENSG00000170469.9;ENSG00000214717.8;ENSG00000265089.1;ENSG00000174586.9;ENSG00000255621.1;ENSG00000229481.2;ENSG00000236972.2;ENSG00000183454.12;ENSG00000214776.8;ENSG00000142082.13;ENSG00000250645.1 | 52 |
| 17_genes | ENSG00000219755.1;ENSG00000281205.1;ENSG00000189171.12;ENSG00000074410.12;ENSG00000074416.12;ENSG00000244245.1;ENSG00000170469.9;ENSG00000214717.8;ENSG00000265089.1;ENSG00000174586.9;ENSG00000255621.1;ENSG00000212864.3;ENSG00000236972.2;ENSG00000183454.12;ENSG00000214776.8;ENSG00000142082.13;ENSG00000250645.1 | 99 |
| 16_genes | ENSG00000219755.1;ENSG00000189171.12;ENSG00000074410.12;ENSG00000074416.12;ENSG00000244245.1;ENSG00000135063.16;ENSG00000214717.8;ENSG00000265089.1;ENSG00000174586.9;ENSG00000255621.1;ENSG00000212864.3;ENSG00000236972.2;ENSG00000183454.12;ENSG00000214776.8;ENSG00000142082.13;ENSG00000250645.1 | 231 |

| | | |
|---|---|---|
| 9_genes | ENSG00000074410.12;ENSG00000151883.15;ENSG00000135063.16;ENSG00000214717.8;ENSG00000265089.1;ENSG00000174586.9;ENSG00000204852.14;ENSG00000214776.8;ENSG00000142082.13 | 333 |
| 12_genes | ENSG00000074410.12;ENSG00000074416.12;ENSG00000151883.15;ENSG00000135063.16;ENSG00000214717.8;ENSG00000265089.1;ENSG00000174586.9;ENSG00000204852.14;ENSG00000236972.2;ENSG00000214776.8;ENSG00000142082.13;ENSG00000100985.7 | 351 |
| 18_genes | ENSG00000219755.1;ENSG00000183578.5;ENSG00000189171.12;ENSG00000074410.12;ENSG00000074416.12;ENSG00000151883.15;ENSG00000135063.16;ENSG00000214717.8;ENSG00000265089.1;ENSG00000174586.9;ENSG00000255621.1;ENSG00000212864.3;ENSG00000204852.14;ENSG00000236972.2;ENSG00000183454.12;ENSG00000214776.8;ENSG00000142082.13;ENSG00000100985.7 | 411 |
| 18_genes | ENSG00000219755.1;ENSG00000189171.12;ENSG00000074410.12;ENSG00000074416.12;ENSG00000151883.15;ENSG00000135063.16;ENSG00000214717.8;ENSG00000265089.1;ENSG00000174586.9;ENSG00000255621.1;ENSG00000212864.3;ENSG00000204852.14;ENSG00000236972.2;ENSG00000183454.12;ENSG00000214776.8;ENSG00000142082.13;ENSG00000100985.7;ENSG00000250645.1 | 418 |
